# Two Cases of Post-Traumatic Mucormycosis due to *Mucor circinelloides*: Salvage Therapy with a Combination of Adjunctive Therapies

**DOI:** 10.1155/2022/4949426

**Published:** 2022-05-04

**Authors:** A. De Paepe, K. Dams, D. Robert, R. Jacobs, G. L. Ten Kate, S. Van Ierssel, H. Jansens, M. Lammens, A. Van Beeck, P. G. Jorens

**Affiliations:** ^1^Departments of Critical Care Medicine, Antwerp University Hospital, University of Antwerp, Edegem, Belgium; ^2^Departments of Infectious Diseases, Antwerp University Hospital, University of Antwerp, Edegem, Belgium; ^3^Departments of Microbiology, Antwerp University Hospital, University of Antwerp, Edegem, Belgium; ^4^Departments of Pathology, Antwerp University Hospital, University of Antwerp, Edegem, Belgium; ^5^Departments of Orthopaedics, Antwerp University Hospital, University of Antwerp, Edegem, Belgium

## Abstract

Mucormycosis is a rare, emerging angioinvasive infection caused by ubiquitous filamentous fungi. In recent decades, an increase in cutaneous or post-traumatic mucormycosis has been reported. We describe two cases of post-traumatic wound infections with *Mucor circinelloides*, a mucor species *only rarely reported as a* cause of post-traumatic mucormycosis. Often considered lethal, management required a combination of medical and surgical therapies to achieve a favorable outcome in both cases.

## 1. Introduction

Mucormycosis is an aggressive fungal disease caused by members of the phylum Glomeromycota, subphyla Mucoromycotina and Entomophtoromycotina (formerly phylum Zygomycota, orders of Mucorales and Entomophthorales) [1–3]. Most frequently, mucormycosis is a disease of an immunocompromised host, either due to hematological malignancy, hematopoietic stem cell or solid organ transplantation, or poorly controlled diabetes mellitus, especially in the presence of diabetic ketoacidosis [4]. More recently, COVID-19 and the associated treatment with corticosteroids were also identified as risk factors for the development of mucormycosis [5]. The disease has various presentations: rhino-orbito-cerebral, pulmonary, cutaneous, gastrointestinal, or disseminated disease. Cutaneous disease, although rare, also occurs in immunocompetent hosts, especially following trauma [6].

In this report, we describe two cases of post-traumatic mucormycosis due to infection with *Mucor circinelloides* in two road traffic accident victims.

## 2. Case 1 and 2

In the first case, the proven invasive fungal disease was confirmed by histopathology (presence of hyphae and necrotic tissue), whereas in the second case diagnosis was based on the clinical picture and tissue culture of bone and muscle. Histopathology showed muscle necrosis without the presence of hyphae [7]. The cultures were sent to the national reference center for invasive fungal disease for identification (matrix-assisted laser desorption/ionization time of flight (MALDI-TOF) and mass spectrometry identification (MSI) platform) and susceptibility testing (*E*-test). This mucor species has rarely been detected in humans after trauma or in cutaneous infections. Effective management required repeated surgical debridement, conventional antifungal therapy, and a combination of adjunctive therapies.

### 2.1. Case 1 Presentation

A 20-year-old, previously healthy male patient suffered from a road traffic accident. He was hit by a truck with a subsequent fall off a bridge and landed in the bushes. During the primary survey, performed by emergency medical services, a traumatic amputation of the right leg above the knee was identified. The patient was stabilized, intubated, mechanically ventilated, and transferred to our hospital. A secondary survey showed the above-knee amputation with a large skin and soft tissue laceration reaching up to the perineum, a communitive right acetabular fracture, replaced pubic fractures, and several thoracic and lumbar spine fractures, including a burst fracture of the 1^st^ lumbar vertebra. The neurological function of the left lower limb was preserved. First-look surgery was performed: ligation of the right femoral artery, suturing of the perineal laceration, and creation of a loop colostomy. The amputation wound was left open and covered with chloramine wound dressings. At this time, the patient had received several red blood cell transfusions. Prophylactic treatment with broad-spectrum intravenous antibiotics (piperacillin-tazobactam) was set according to local and international guidelines, and the patient was transferred to the intensive care unit (ICU) for further treatment.

On Day 1 after hospital admission, the patient developed septic shock. Necrosis was observed in the amputation wound, both at the level of the superficial and deep soft tissues. Large amounts of wound exudate drained from the amputation wound. The patient was sent to the operating room for surgical debridement, and adjunctive treatment with clindamycin was added for a presumed necrotizing skin and soft tissue infection. Cultures of operatively obtained tissue samples yielded multidrug-resistant *Escherichia coli* and *Enterococcus faecium*. The patient was switched to an antibiotic regimen of meropenem and vancomycin. Despite repeated surgical debridement, the necrotic skin and soft tissue infection rapidly progressed. Given the availability of hyperbaric oxygen therapy (HBOT) in our center, six sessions of two hours, 2 atmospheres, while sedated and mechanically ventilated, were performed, which effectively maintained progressive skin and soft tissue necrosis.

**Figure 1:**
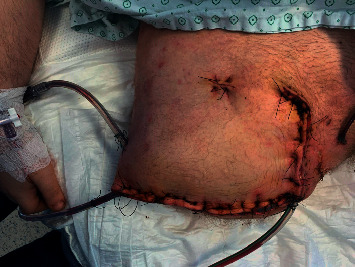
Partially closed amputation stump with drains in place (first case).

**Figure 2:**
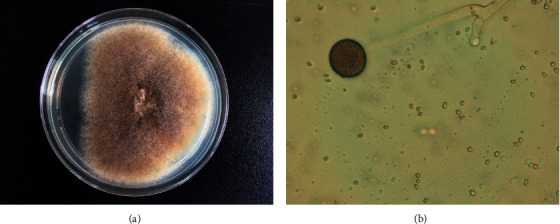
(a) Gray-brownish colony growth on Sabouraud dextrose agar. (b) Identification under a microscope in the lab (first case). Sporangium with spores on a branched sporangiophore.

On Day 9 after the patient was injured, an attempt was made to partially close the wound (Figure 1) after another surgical debridement, given the favorable clinical evolution with HBOT. However, cultures that were taken during this surgery from the gluteal degloved zone showed the presence of fungi, which were later identified as *Mucor circinelloides* (Figures 2(a) and 2(b)). An antifungigram is presented in Table 1. Antifungal therapy with systemic liposomal amphotericin B at 5 mg/kg body weight/day from Day 14. The same day, the patient underwent embolization for arterial bleeding of the right deep femoral artery. The hematoma that was formed due to the arterial bleeding was drained after adequate bleeding control was achieved (Day 15). Wound dressings with povidone-iodine were used from that time onward.

**Figure 3:**
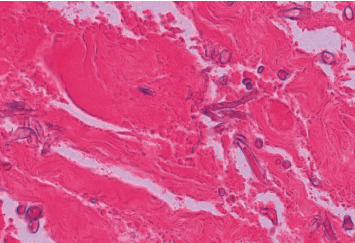
Hematoxylin-eosin staining of tissue obtained from the post-traumatic stump on Day 24 after the trauma (first case). *M circinelloides* dimorphic phases with pauci-septated wide hyphae with right-angle branching are seen. Please note the absence of infiltration of the underlying muscle with inflammatory cells, in devitalised tissue.

Repeated debridement yielded persistence of positive tissue cultures with *Mucor circinelloides* and regional progression of superficial and deep soft tissue necrosis and perineal cellulitis. Given the lack of clinical improvement, adjunctive therapies were added on Day 23: systemic anidulafungin (100 mg daily for 20 days) and local conventional amphotericin B wound dressings (75 mg =1 mg/kg body weight) in 500 ml (0.15 mg/ml) twice daily for 30 minutes. Histopathology of the resected specimen on Day 24 showed necrotic tissue with the presence of large hyphae (Figure 3). Amphotericin B wound dressings were continued for 5 days and replaced by negative pressure wound therapy (vacuum-assisted closure). With this combination therapy, a clinical improvement of the wound was achieved, and cultures of the wound became negative for fungi on Day 37 after the trauma.

Antibiotic therapy was de-escalated to ciprofloxacin and amoxicillin (guided by antibiogram) on Day 26 and continued for a total duration of six weeks for suspected but undocumented osteomyelitis. Antifungal therapy with systemic liposomal amphotericin B was continued for 49 days until almost complete closing of the wound. The patient developed hypokalemia, but no further systemic toxicity of amphotericin B and/or other antimicrobials was observed.

There was no documented systemic dissemination of *Mucor circinelloides*, and blood cultures remained negative. Spine surgery (lumbar fusion) was deferred until significant infection control was achieved to lower the risk of surgical site infection on spinal instrumentation.

The patient was discharged from the ICU to the trauma ward on Day 42 after admission. He left the hospital for a rehabilitation center 35 days later. His further course was uneventful.

### 2.2. Case 2 Presentation

We report another case of a 41-year-old male patient with a history of depression who was hit by a truck while riding a bicycle. The primary survey showed traumatic amputation of both the lower legs and the right forearm with severe degloving injuries and a large scalp wound. He was intubated on-site and transported to our emergency department. The secondary survey showed the following additional injuries: right-sided temporal skull fracture without signs of intracranial hemorrhage, nonreplaced orbital fracture, minimal right-sided pneumothorax, and right clavicle and scapula fracture. A damage control surgery was performed: disarticulation at the level of the left knee, above the knee amputation of the right leg, and right forearm amputation. The soft tissues were approximated and covered with wound dressings. The scalp wound was surgically rinsed and covered with wound dressings. Other injuries were treated conservatively. Stabilization of hemorrhagic shock required multiple transfusions and the temporary use of an intra-aortic balloon catheter to support hemorrhagic control. Empiric antibacterial therapy with piperacillin-tazobactam was started, and the patient was transferred to the ICU for further treatment.

Second-look surgery was performed on Day 2 for the three amputation wounds. The soft tissues in the three amputation wounds were mostly viable, debridement was performed, and the wounds were closed. The patient was weaned from the ventilator on Day 3. In the following days, progressive skin and soft tissue necrosis were observed near the amputation sites of the right leg and arm. Repeat debridement was performed on Day 9. The necrotic skin of the right arm was also debrided, and the wound was rinsed and covered with chloramine wound dressings. Debridement of the muscles of the right upper leg was performed, and the wound was closed again. Specimens collected at the right leg amputation wound (including bone marrow cultures) on Day 9 showed growth of *Enterococcus faecium* and Mucorales, which were later identified as *Mucor circinelloides* (antifungigram is presented in Table 1). Specimens taken from the right arm amputation wound showed growth of *Bacillus cereus* and *Mucor circinelloides*, after earlier specimens had not shown growth of fungi. On Day 10, the patient was started on systemic liposomal amphotericin B (5 mg/kg body weight/day) for mucormycosis and vancomycin for osteomyelitis. Treatment with systemic liposomal amphotericin B was continued for a total duration of 49 days. The medical treatment was complicated by acute kidney injury that did not require dialysis.

Additional surgical debridement of the right arm and right leg amputation wounds was performed on Day 11, negative pressure wound therapy (vacuum-assisted closure) was started, and several debridements were performed in the following weeks. The first negative fungal culture of the right arm was 4 days after the initiation of systemic liposomal amphotericin B.

The first negative fungal culture of the right leg was 14 days after initiation of amphotericin B, and this wound also required repeated debridement, including abscess drainage (*Enterobacter cloacae*) and vacuum-assisted wound closure. Despite favorable evolution of the wound, the patient developed osteomyelitis of the right femur with *Enterobacter cloacae*, for which he was treated with tigecycline, followed by oral ciprofloxacin for a total duration of 6 weeks.

The amputation wound of the left leg required a gracilis flap, which failed, and an amputation above the knee amputation was performed. In the following weeks, regular debridement and continuous vacuum-assisted wound closure therapy were required for the wound on the left leg.

The patient was discharged from the ICU to the trauma ward on Day 16 after admission. He left the hospital for a rehabilitation center 57 days later. He remains in outpatient follow-up for the disability following trauma and a post-traumatic adjustment disorder.

## 3. Discussion

Mucormycosis is a potentially lethal fungal infection caused by members of the phylum *Glomeromycota,* subphyla *Mucoromycotina* and *Entomophthorales*. In addition to *M. circinelloides*, the genus *Mucor* contains 11 other clinically relevant species [8]. *M. circinelloides* is a saprophytic fungus that can be found worldwide in soil and decaying organic material. *Mucor* spores are not dispersed by air, but theoretically, they can be transmitted through direct contact [9, 10]. Despite this transmission, *Mucor* species are uncommon in trauma. *Rhizopus, Apophysomyces, Saksenaea,* and *Lichtheimia* species are more frequently reported as causative pathogens in trauma-related mucormycosis [11]. Human infections with *M. circinelloides* are rare but are probably underestimated by a lack of differentiation up to the species level in the past.

A review of the existing literature shows that *Mucor circinelloides* is responsible for causing food-related outbreaks of paronychia in orange workers [12] and gastrointestinal mucormycosis due to yoghurt contamination [13].  Table 2 provides an overview of all cases of mucormycosis due to *M. circinelloides* in humans that occurred after minor or major trauma [14–20].

*M. circinelloides* was also implicated in an outbreak of 6 cases in a burn unit, and an environmental source was suspected [16]. Furthermore, we found two cases of cutaneous mucormycosis with *M. circinelloides* in immunocompromised patients after minor injury or no injury at all [15, 21] and one case of secondary cutaneous mucormycosis with *M. circinelloides* in the context of disseminated disease [22]. Trauma-related mucormycosis due to *M. circinelloides* has only rarely been reported in the literature. One case was found after minor trauma in an immunocompromised (hematological malignancy) patient [14]. Major trauma-related mucormycosis with *M. circinelloides*, such as our case, was reported in only two other publications (3 cases) [17, 18].

Most cases of mucormycosis occur in immunocompromised (hematological malignancy, hematopoietic stem cell transplantation, solid organ transplantation) or diabetic patients, particularly in the presence of diabetic ketoacidosis. During the COVID-19 pandemic, in India and to a lesser extent in other countries, several authors reported cases of rhino-orbito-cerebral and pulmonary mucormycosis in patients with COVID-19 [23, 24]. Classic mucormycosis risk factors apply in patients with COVID-19 (poorly controlled diabetes and systemic corticosteroids). On the other hand, cutaneous and soft tissue mucormycosis may occur in immunocompetent hosts because of trauma, but mainly in European countries. In the European immunocompetent host, cutaneous mucormycosis is most prevalent, while in Iran and India rhino-orbitocerbral disease is the most prevalent in these patients [25, 26], the reason for this difference is not understood. Whether these differences represent climatologic, routine practice, patient-specific differences, or an indirect association with diagnostic and therapeutic resources is not yet understood and forms a target for further investigation. In developing countries and lower-resource settings, the diagnosis of mucormycosis might be more challenging given the unavailability of fungal cultures.

Traumas associated with mucormycosis are natural disasters, motor vehicle accidents, explosions, surgery, and burns [3]. The infection can be localized to the skin (4–10% mortality) or with extension to the deeper soft tissues, muscles, and bones (26–43% mortality). In rare cases, the disease may disseminate from a primary cutaneous site [27]. Whereas mortality in trauma-related mucormycosis ranges between 25–41%, mortality ranges from 40% to 80% in other presentations of mucormycosis. Patients with hematological malignancy, hematopoietic stem cell transplantation and extensive burns have the worst prognosis [3]. Patients with trauma-related mucormycosis are often younger and have sustained traffic accidents, agriculture-related accidents, or natural disasters. Infected wounds show signs of necrosis, cellulitis, indurated plaques, nodules, purpuric lesions, and ulceration. Other clinical signs are fever and leukocytosis. Risk factors for trauma-related mucormycosis are leg amputation above the knee, large wounds, rhabdomyolysis, and penetration injuries [28].

Infection in post-traumatic mucormycosis occurs following direct inoculation of soil-borne spores. Introduction of the spores in deep soft tissues is followed by germination of the spores and formation of hyphae [11]. Hyphae formation and proliferation are promoted by a decreased local pH level, which causes the release of iron from transferrin, decreased chemotaxis, and functional impairment of neutrophils due to trauma-associated immune dysregulation [4]. As Mucorales require iron for growth [29, 30], it is tempting to state that the bleeding complication in the first case on Day 9 may have contributed further to the disease progression in the following days [4, 28, 31]. The growth of hyphae causes destruction of tissue and angioinvasion, which may cause thrombosis of blood vessels, which in turn causes necrosis [28]. In the first case, the trauma and subsequent ligation of the femoral artery probably initiated necrosis. Progressive necrosis, despite broad-spectrum antibiotic therapy, may have contributed to the growth of *M. circinelloides*, but its angioinvasive properties may also have contributed to the observed progressive tissue necrosis.

Current methods to diagnose mucormycosis include culture of infected tissue on selective media such as Sabouraud dextrose agar. Fungal hyphae can also be observed on hematoxylin-eosin staining, where *Mucoromycotina* appear as non- or oligoseptated hyphae with irregular branching at 90° angles [32]. Diagnosis is often delayed to approximately 2 weeks after trauma, which may be partly attributed to the current diagnostic methods [18]. Data from military trauma [28] show that frozen sections during surgical debridement may play a role in the rapid diagnosis of invasive fungal infections. In high-risk patients (lower limb amputation, extensive necrosis, and extensive wound contamination), frozen sections may shorten the delay to diagnosis. Molecular diagnostic methods such as polymerase chain reaction (PCR) can be used to detect Mucorales in tissue samples. PCR has also been under investigation as a noninvasive diagnostic tool by using blood samples in patients with suspected pulmonary mucormycosis and burn victims, potentially opening a window of opportunity for preemptive therapy or at least proactive surgical debridement when the patient is not yet clinically deteriorating [3]. Specific serological biomarkers are currently not available. In the presented cases, culture and histopathologic examination of the early tissue samples did not show fungal infection, and frozen sections were not used in these cases. During the following surgical debridement, resected tissue samples from soft tissue and/or bone that was clinically suspected for infection were sent for bacteriological and mycological culture. Molecular diagnostic tools (PCR) for the detection of Mucorales are not available in our center. Coinfection with bacterial pathogens is common [28], and coinfection with other fungi may also occur [6].

In the management of post-traumatic mucormycosis, early, extensive, and repeated surgical debridement is paramount, and amputation is often needed, resulting in long-term disability [28]. However, surgical debridement improves outcomes when added to antifungal therapy [33]. The preferred systemic antifungal therapy is liposomal amphotericin B (5 mg/kg/day). In renal disease or in cases of intolerance, treatment with isavuconazole (200 mg three times daily for 2 days, followed by 200 mg once daily from Day 3) or posaconazole (300 mg twice daily on Day 1, followed by 300 mg once daily from Day 2) can be considered if the infecting fungus is susceptible. In progressive disease, liposomal amphotericin B dosing can be maximized to 10 mg/kg/day, or isavuconazole can be used as salvage therapy [3]. In trauma-related mucormycosis with extensive necrosis, inadequate penetration in the infected tissue of systemically administered antifungals can be considered. There is no clear directive for treatment duration, and it is advised to continue systemic antifungal therapy until clinical and/or radiological resolution [3]. The duration of antifungal therapy reported in the literature varies from 23 to 55 days [11, 18, 28].

During the disease course, we used several adjunctive therapies in the first case, which led to a combined therapeutic approach that positively altered the disease course. Anidulafungin, while generally not active against Mucorales, has been shown to induce synergy in combination with amphotericin B for the treatment of mucormycosis in vitro and in mice [34, 35]. Other combinations of antifungals (amphotericin B in combination with caspofungin, micafungin, posaconazole, or itraconazole) were insufficient to improve outcomes [33]. Given the extensive necrosis of the wounds and uncertainty as to whether adequate penetration of intravenous drugs was achieved, we applied amphotericin B wound dressings in the first case, as is sometimes performed in rhino-orbital disease [36] and in post-traumatic mucormycosis [17, 18]. HBOT was used in the first case prior to the diagnosis of mucormycosis. Although unproven, it may have some promising antifungal effects by reversal of tissue hypoxia, normalization of tissue pH levels, and restoration of phagocytic function [6, 28, 37]. It is possible that the early use of HBOT initially reversed progression of mucormycosis. Initial clinical improvement was observed, hence the partial closing of the amputation stump. However, cessation of HBOT and bleeding in the amputation stump may have promoted fungal growth. The use of negative pressure wound therapy (vacuum-assisted wound closure) is recommended for wound management in mucormycosis and was started after progressive necrosis was halted in the first case and early in the second case [38].

## 4. Conclusion

Apart from detection in humans after burns, these two cases represent one of few documented cases of *M. circinelloides* isolation in post-traumatic tissue in immunocompetent patients. Post-traumatic mucormycosis is a challenging disease, and the detection of this fungus and the use of several adjunctive therapies were crucial for the favorable outcome: surgical excision, intravenously administered liposomal amphotericin B, hyperbaric oxygen, intravenous anidulafungin, and local amphotericin B. The use of repeated surgical debridement, tissue cultures, and histopathological examination provided timely diagnosis and follow-up after initiation of antifungal therapy, and perioperative frozen-section margins can provide guidance to surgeons during surgical debridement. The use of rapid diagnostic tests, if available, might be of use to further expedite diagnosis in patients with a suspected clinical picture. Given the ubiquity of Mucorales in nature and the absence of known risk factors in patients with post-traumatic mucormycosis, prevention of this devastating disease can be achieved by reducing severe road traffic accidents. Awareness that invasive fungal disease may develop in these patients is paramount for appropriate medical care. Although generally not considered a contagious disease, hospitals should be vigilant for outbreaks [16].

## Figures and Tables

**Table 1:** Antifungigram of *M. circinelloides* in both cases.

Antifungal agent	MIC in *μ*g/ml, first case	MIC in *μ*g/ml, second case
Amphotericin B	0.06	0.06
Itraconazole	>16	2
Voriconazole	>16	>16
Posaconazole	2	>16

MIC, minimal inhibitory concentration.

**Table 2:** Characteristic findings of previously reported cases of mucormycosis caused by *Mucor circinelloides* after trauma or burns.

Case number	Author	Age/sex	Mechanism of trauma	Injury	Underlying predisposing condition	Diagnosis (trauma = D0)	Treatment	Outcome
1	De Paepe et al.	20/male	Road traffic accident	Traumatic amputation of right leg above the knee perineal laceration	None	D14	Surgical debridement, systemic liposomal amphotericin B, HBOT, anidulafungin, local amphotericin B, vacuum-assisted wound closing	Recovered
2	De Paepe et al.	41/male	Road traffic accident	Traumatic amputation of three limbs	None	D10	Surgical debridement, amputation, systemic amphotericin B, vacuum-assisted wound closing	Recovered
3	Holoubek et al. [17]	50/male	Road traffic accident	Subluxation left ankle	None	D24 (D32 Diagnosis of *Fusarium sp.)*	Surgical debridement, systemic voriconazole, local amphotericin B, vacuum-assisted wound closing	Recovered
4	Schaal et al. [19]	29/N/A	Burn	61% TBSA/52% FTBSA	N/A	2.5 weeks	Systemic amphotericin B, local amphotericin B	Death
5	Schaal et al. [19]	33/N/A	Burn	40% TBSA /30% FTBSA	N/A	4 weeks	Local amphotericin B	Recovered
6	Sekowska et al. [20]	26/male	Road traffic accident	Open knee dislocation, multiple fragment fractures of the left leg	N/A	D3	Systemic amphotericin B, HBOT, amputation	Recovered
7	Chandra et al. [14]	62/female	Minor abrasion	Necrotic ulcer in forearm	Myelodysplastic syndrome	4 weeks	Surgical excision	No recurrence, death from hematological malignancy
8	Fingeroth et al. [15]	23/female	Insect bite	Necrotic lesion	Acute monocytic leukemia	D8	Amphotericin B	Recovered
9–18	Garcia-Hermoso et al. [16]	N/A	Burn	Burn wounds	N/A	N/A	N/A	3 recovered 7 deaths
19-20	Lelievre et al. [18]	Median age 42.9/68.7% male	N/A	Trauma 81.2% involvement of limbs	Underlying disease in 31.2%	Median D15.5	81.2% antifungal therapy,, 93.7% surgery, 18.7% local therapy, 18.7% vacuum-assisted wound closing	37.5% death

TBSA, total body surface area burned; FTBSA, full-thickness burn surface area; N/A, not available; HBOT, hyperbaric oxygen therapy; D, day.

## Data Availability

All data used to support the findings of this study are available from the corresponding author upon request.
